# Evolution of Network Biomarkers from Early to Late Stage Bladder Cancer Samples

**DOI:** 10.1155/2014/159078

**Published:** 2014-09-18

**Authors:** Yung-Hao Wong, Cheng-Wei Li, Bor-Sen Chen

**Affiliations:** Lab of Control and Systems Biology, Department of Electrical Engineering, National Tsing Hua University, Hsinchu 30013, Taiwan

## Abstract

We use a systems biology approach to construct protein-protein interaction networks (PPINs) for early and late stage bladder cancer. By comparing the networks of these two stages, we find that both networks showed very significantly different mechanisms. To obtain the differential network structures between cancer and noncancer PPINs, we constructed cancer PPIN and noncancer PPIN network structures for the two bladder cancer stages using microarray data from cancer cells and their adjacent noncancer cells, respectively. With their carcinogenesis relevance values (CRVs), we identified 152 and 50 significant proteins and their PPI networks (network markers) for early and late stage bladder cancer by statistical assessment. To investigate the evolution of network biomarkers in the carcinogenesis process, primary pathway analysis showed that the significant pathways of early stage bladder cancer are related to ordinary cancer mechanisms, while the ribosome pathway and spliceosome pathway are most important for late stage bladder cancer. Their only intersection is the ubiquitin mediated proteolysis pathway in the whole stage of bladder cancer. The evolution of network biomarkers from early to late stage can reveal the carcinogenesis of bladder cancer. The findings in this study are new clues specific to this study and give us a direction for targeted cancer therapy, and it should be validated in vivo or in vitro in the future.

## 1. Introduction

Cancer is the leading cause of death worldwide and its etiology occurs at the DNA, RNA, or protein level. It is a very complex disease involving cascades of spatial and temporal changes in the genetic network and metabolic pathways [1]. Various research studies have revealed that cancers are caused by multiple factors and intertwined events. Thus, in cancer therapy, it is important to dissect the diverse molecular mechanisms of cancer to identify potential cancers. Bladder cancer is amongst the 10 most common carcinomas in the USA, with 72,570 newly diagnosed cases, and it was the cause of 15,120 deaths in 2013 [2]. In particular, Kaufman et al. pointed out to it as the second most common form of cancer in 2008 [3]. In this study, we compared the early and late stages of bladder cancer to reveal additional mechanisms of bladder cancer development [4].

Biomarker discovery of various cancers is one of the key topic areas of cancer research. It can aid investigations into carcinogenesis and novel drug designs for cancer therapy. Several bioinformatics methods have been developed and applied to compare normal tissue with cancerous tissue to determine what cancer driving genes can act as cancer biomarkers [5–12].

Genes and proteins function cooperatively to regulate common biological cell processes by coregulating each other [13]. Generally, molecular regulation and interaction proceed with time and vary in different tissues. There must exist great differences in these variations between cancer and normal tissue. Proteins mutually interact with each other in the cell, and they form the PPI networks (PPINs). Currently, a lot of the research has focused on the relationship between PPINs and cancer development. For example, analysis of the cancer-related PPINs of apoptosis has unraveled the molecular mechanisms of cancer, which has helped to identify potential novel drug targets [14]. Our previous work [14] had successfully identified the network markers of lung cancer. In this study, we modified our previous method and applied the novel concept to study the evolution of network markers from early to late stage bladder cancer.

Based on their PPI information and the gene expression profiles from cancer and surrounding normal samples, two PPI networks with quantitative protein association abilities for each cancer stage (early stage and late stage) and the surrounding noncancerous tissue are constructed, respectively. For each stage, the network structure and protein association abilities of the cancer and noncancer PPI networks are then compared to obtain sets of significant proteins which play important roles in the carcinogenesis process of bladder cancer.

Recently, PPI targets seem to have become a paradigm for the drug discovery of cancer therapy and precision medicine [15]. Unlike conventional drug design focusing on the inhibition of a single protein, usually an enzyme or receptor, small-molecule inhibition of direct PPIs that mediate many important biological processes is an emerging and challenging concept in drug design, especially for cancer. Extensive biological and clinical investigations have led to the identification of PPI hubs and nodes that have been critical for the acquisition and maintenance of characteristics for cell transformation in cancer. Such cancer-enabling PPIs will become promising therapeutic targets in anticancer strategies as the technologies in PPI modulator discovery and validating agents in the clinical setting advance in the future [15].

Therefore, future research directed at PPI target discovery, PPI interface characterization, and PPI-focused chemical libraries are expected to accelerate the development of the next generation of PPI-based anticancer agents. However, the PPI networks of cancer are very complex and quite differ between early and late stage cancer. In such circumstances, we will focus on the PPI network markers with their significant carcinogenesis relevance value (CRV) to exploit the important targets and their PPI interface for early and late stage cancer characterization. Then, we will not only gain insight into the crucial common pathways involved in bladder carcinogenesis, but we will also obtain a highly promising PPI target for bladder cancers. If we are then able to develop various combined anticancer strategies to target PPIs in the early and late stage network markers in the future, it may provide emerging opportunities for anticancer therapeutic approaches.

Chen et al. developed a dynamical network biomarker (DNB) that can serve as a general early warning signal to indicate an imminent bifurcation or sudden deterioration before the critical transition occurs; that means it can identify predisease state by time series microarray data. We use different approach from their methods by sample microarray data from bladder cancer patients of different stages. Our approach could also be extended to predict some similar results as their research. That is, in this study, we simply divided the cancer into early and late stages, but there are more stages of cancer, such as stages I, II, III, and IV. If we could observe the time evolution of the cancer biomarkers at these more different stages, we could also predict the predisease state by comparing it with these cancer biomarkers at different stages [16–18].

## 2. Materials and Methods

### 2.1. Overview of the Bladder Cancer Network Markers Construction Process

**Figure 1:**
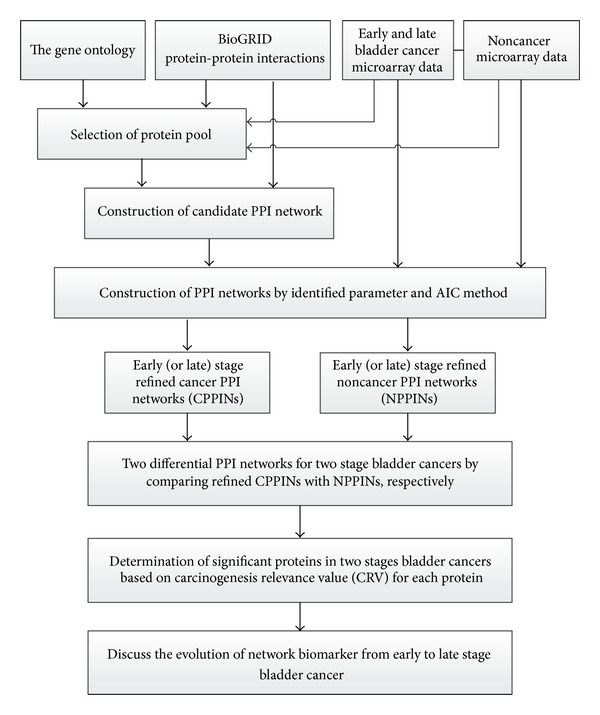
The flowchart of constructing both stages of network marker of bladder cancer and the investigation of the carcinogenesis mechanisms. We integrate microarray data, GO database, and PPI information to construct the PPI network. These data are used for pool selection, and then the selected proteins and the microarray data are used for the contribution of protein-protein interaction network (PPIN) by maximum likelihood estimation and model order detection method, resulting in bladder cancer PPIN (CPPIN) and noncancer PPIN (NPPIN) of early and late stage. The two constructed PPINs can be used for the determination of significant proteins of tumorigenesis by the difference between two PPI matrices of two constructed PPINs. With the help of the differential PPI matrix (network) between CPPIN and NPPIN, carcinogenesis relevance value (CRV) is computed for each protein, and significant proteins in carcinogenesis are determined based on* P* value the CRVs of these proteins in the differential PPI matrix between CPPIN and NPPIN. These significant proteins are obtained for early and late stage bladder cancers.

A flowchart representing the construction of network biomarkers for early and late stage bladder cancer is shown in Figure 1.   We combined two data sources: (1) microarray data of bladder cancer and noncancer samples from the GEO database, while the cancer samples were divided into two groups: early stage and late stage bladder cancer. (2) The PPI database was required to construct the PPINs for bladder cancer. This data was used for PPI pool selection and the selected PPIs and the microarray data were then used for PPI network (PPIN) construction. Through regression modeling and the maximum likelihood parameter estimation method, a cancer PPIN (CPPIN) and a noncancer PPIN (NPPIN) was then obtained. The two constructed cancer and noncancer PPINs were compared to obtain the sets of significant proteins for bladder cancer based on the carcinogenesis relevance value (CRV) for each protein and the statistical assessment. The significant proteins and PPIs within these proteins were used to construct network markers at early and late stage bladder cancer.

### 2.2. Data Selection and Preprocessing

The microarray gene expression dataset of bladder cancer was obtained from the NCBI gene expression omnibus (GEO) [19]. In this study, we chose GSE13507 [20] and its corresponding platform GPL6012 as our research object. The same dataset contained the early and late stage bladder cancer and noncancer samples. We only used the data derived from nonprocessed primary biopsies to avoid the discrepancies in gene expression that are intrinsic to cell culture and fixation. Therefore, the dataset utilized contained primary tumor samples of both stages from patients and adjacent nontumor tissue samples from the same cancer patients, which could be considered as control samples. To describe the extent of a patient's cancer, the cancers were classified into four stages according to their degree of invasion and migration using the TNM staging system, as defined by the American Joint Committee on Cancer (AJCC) and the International Union against Cancer (UICC). We then divided the cancer samples into two groups. In general, stages I and II described early stage cancers that have higher curability rates with medical treatment, while stages III and IV described the late stages. However, there were no corresponding noncancer samples in the surrounding area for each stage and we had only one group of surrounding noncancer samples (Table 1). We built CPPIN and NPPIN for both early and late stage bladder cancer in this study. We obtained 37 and 106 samples for the early and late stage cancer, respectively, and 58 noncancer samples. To avoid overfitting in network construction, the maximum degree of the proteins in the PPI network should be less than the cancer/noncancer sample number [14]. In this dataset, we had a greater number of cancer and noncancer samples to overcome the sample size restriction on the size of the network. Prior to further analysis, the gene expression value, *h*_*ij*_, was normalized to *z*-transformed scores, *g*_*ij*_, for each gene,* i*, and then the normalized expression value resulting had a mean *μ*_*i*_ = 0 and standard deviation *σ*_*i*_ = 1 over sample *j* [11, 14].

The PPI data for* Homo sapiens* were extracted from the Biological General Repository for Interaction Database (BioGRID, downloaded in October 2012). BioGRID is an open-access archive of genetic and protein interactions that are curated from the primary biomedical literature of all major model organisms. As of September 2012, BioGRID houses more than 500,000 manually annotated interactions from more than 30 model organisms [21]. The above two databases were mined for bladder cancer and noncancer PPI networks using their corresponding microarray data. These early and late stage bladder cancer and noncancer PPI networks were then compared to obtain network markers.

### 2.3. Selection of Protein Pool and Identification of the Protein-Protein Interaction Networks (PPINs) for Cancerous and Noncancerous Cells

To integrate gene expression with PPI data to construct the corresponding CPPINs and NPPINs, we set up a protein pool containing differentially expressed proteins. The gene expression values were reasonably assumed to correlate with protein expression levels. We used one-way analysis of variance (ANOVA) to analyze the expression of each protein and select for proteins with differential expression levels. This method allowed determination of significant differences between cancer and noncancer datasets. The null hypothesis (Ho) was based on the assumption that the mean protein expression levels of cancer and noncancer sets are the same. Bonferroni adjustment [22], a type of multiple testing, was used to detect and correct proteins with discrepancy. Proteins with a *P* value of less than 0.01 were included in the protein pool. However, if the proteins in the protein pool did not have PPI information, they were eliminated. In addition, proteins that were not already in the protein pool were included if their PPI information could determine that they had a tight relationship with proteins already in the pool. As a result, the protein pool contained proteins that had certain differences in expression levels and proteins that had tight relationships with the aforementioned proteins. In this case, the protein pool in bladder cancer consisted of 2,245 proteins in the early stage and 1,101 proteins in the late stage.

On the strength of the significant pool and PPI information, candidate PPI networks for early and late stage bladder cancer were constructed for bladder cancer and noncancer by linking the proteins that interacted with each other. In other words, the proteins that had PPI information through the pool were linked together, resulting in candidate PPI networks.

As the candidate PPIN included all possible PPIs under various environments, different organisms, and experimental conditions, the candidate PPIN needed to be further confirmed by microarray data to identify appropriate PPIs according to the biological processes that are relevant to cancer. To remove false positive PPIs from each candidate PPIN for different biological conditions, we used both a PPI model and a model order detection method to prune each candidate PPIN using the corresponding microarray data to approach the actual PPIN. Here, the PPIs of a target protein *i* in the candidate PPIN can be depicted by the following protein association model:

(1) $$\begin{array}{c} x_{i}\left[n\right]=\overset{M_{i}}{\underset{j=1}{\sum }}\alpha_{ij}x_{j}\left[n\right]+\omega_{i}\left[n\right], \end{array}$$

where *x*_*i*_[*n*] represents the expression levels of the target protein *i* for the sample *n*; *x*_*j*_[*n*] represents the expression level of the *j*th protein interacting with the target protein *i* for the sample *n*; *α*_*ij*_ denotes the association interaction ability between the target protein *i* and its *j*th interactive protein; *M*_*i*_ represents the number of proteins interacting with the target protein *i*; and *ω*_*i*_[*n*] represents the stochastic noise due to other factors or model uncertainty. The biological meaning of (1) is that the expression levels of the target protein *i* are associated with the expression levels of the proteins interacting with it. Consequently, a protein association (interaction) model for each protein in the protein pool can be built as (1).

After constructing (1) for the PPI model of each protein in the candidate PPIN, we used the maximum likelihood estimation method [23] to identify the association parameters in (1) by microarray data as follows (see Supplementary Materials S.1 available online at http://dx.doi.org/10.1155/2014/159078):

(2) $$\begin{array}{c} x_{i}\left(n\right)=\overset{M_{i}}{\underset{j=1}{\sum }}{\hat{\alpha }}_{ij}x_{j}\left(n\right)+w_{i}\left(n\right), \end{array}$$

where ${\hat{\alpha }}_{ij}$ is identified using microarray data in accordance with the maximum likelihood estimation method (see Supplementary Materials).

Once the association parameters for all proteins in the candidate PPI network were identified for each protein, the significant protein associations were determined using the interaction model order detection method based on the estimated association abilities. The Akaike information criterion (AIC) [23] and Student's *t*-test [24] were employed for both model order selection and significance determination of the protein associations in ${\hat{\alpha }}_{ij}$ (see Supplementary Materials S.2).

### 2.4. Determination of Significant Proteins and Their Network Structures in the Carcinogenesis of Four Types of Cancers

After *P* values were determined using the AIC order detection and Student's *t*-test, spurious false positive PPIs ${\hat{\alpha }}_{ij}$ in (2) were pruned away and only the significant PPIs that remained were refined as follows:

(3) $$\begin{array}{c} x_{i}\left(n\right)=\overset{M_{i}^{\prime }}{\underset{j=1}{\sum }}{\hat{\alpha }}_{ij}x_{j}\left(n\right)+w_{i}^{\prime }\left(n\right),\;i=1,2\ldots M, \end{array}$$

where *M*_*i*_′ ≤ *M*_*i*_ denotes the number of significant PPIs of PPIN, with the target protein *i*. In other words, a number of *M*_*i*_ − *M*_*i*_′ (or false positives) are pruned in the PPIs of target protein *i*. One protein by one protein (i.e., *i* = 1,2,…, *M* for all proteins in the refined PPIN in (3)) results in the following refined PPIN:

(4) $$\begin{array}{c} X\left(n\right)=AX\left(n\right)+w\left(n\right)\\ X\left(n\right)=\left[\begin{array}{c} x_{1}\left(n\right)\\ x_{2}\left(n\right)\\ \vdots \\ x_{M}\left(n\right) \end{array}\right],\;\;A=\left[\begin{array}{ccc} {\hat{\alpha }}_{11} & \ldots & {\hat{\alpha }}_{1M}\\ \vdots & \ddots & \vdots \\ {\hat{\alpha }}_{M1} & \cdots & {\hat{\alpha }}_{MM} \end{array}\right],\\ w\left(n\right)=\left[\begin{array}{c} w_{1}^{\prime }\left(n\right)\\ w_{2}^{\prime }\left(n\right)\\ \vdots \\ w_{M}^{\prime }\left(n\right) \end{array}\right], \end{array}$$

where the interaction matrix *A* denotes the PPIs.

If there is no PPI between proteins *i* and *j* or it is pruned away by AIC order detection due to insignificance in the refined PPIN then ${\hat{\alpha }}_{ij}=0$. In general, ${\hat{\alpha }}_{ij}={\hat{\alpha }}_{ji}$, but if this is not the case, the larger one will be chosen as ${\hat{\alpha }}_{ij}={\hat{\alpha }}_{ji}$ to avoid the situation where ${\hat{\alpha }}_{ij}\neq {\hat{\alpha }}_{ji}$. The above PPIN construction method was employed to construct the refined PPINs for each stage of bladder cancer (early and late) and noncancer cells. The interaction matrices *A* of the refined PPINs in (4) for cancer and noncancer cells of both the early and late stages of bladder cancer were constructed, respectively, as follows:

(5) $$\begin{array}{c} A_{C}^{k}=\left[\begin{array}{ccc} {\hat{\alpha }}_{11,C}^{k} & \ldots & {\hat{\alpha }}_{1M,C}^{k}\\ \vdots & \ddots & \vdots \\ {\hat{\alpha }}_{M1,C}^{k} & \cdots & {\hat{\alpha }}_{MM,C}^{k} \end{array}\right],\\ A_{N}^{k}=\left[\begin{array}{ccc} {\hat{\alpha }}_{11,N}^{k} & \ldots & {\hat{\alpha }}_{1M,N}^{k}\\ \vdots & \ddots & \vdots \\ {\hat{\alpha }}_{M1,N}^{k} & \cdots & {\hat{\alpha }}_{MM,N}^{k} \end{array}\right], \end{array}$$

where *k* = early and late stage bladder cancer; *A*_*C*_^*k*^ and *A*_*N*_^*k*^ denote the interaction matrices of refined PPIN of the *k*th cancer and noncancer, respectively; *M* is the number of proteins in the refined PPIN. Therefore, the protein association model for CPPIN and NPPIN in the *k*th stage bladder cancer and noncancer can be represented by the following equations according to (4) and (5):

(6) $$\begin{array}{c} x_{C}^{k}\left(n\right)=A_{C}^{k}x_{C}\left(n\right)+w_{C}^{k}\left(n\right),\\ x_{N}^{k}\left(n\right)=A_{N}^{k}x_{N}\left(n\right)+w_{N}^{k}\left(n\right), \end{array}$$

where *k* = early and late stage bladder cancer;

(7) $$\begin{array}{c} x_{C}^{k}\left(n\right)={\left[\begin{array}{cccc} x_{1C}^{k} & x_{2C}^{k} & \cdots & x_{MC}^{k} \end{array}\right]}^{T},\\ x_{N}^{k}\left(n\right)={\left[\begin{array}{cccc} x_{1N}^{k} & x_{2N}^{k} & \cdots & x_{MN}^{k} \end{array}\right]}^{T} \end{array}$$

denote the vectors of expression levels; and *w*_*C*_^*K*^(*n*) and *w*_*N*_^*K*^(*n*) indicate the noise vectors of PPINs in the *k*th cancer and noncancer cells, respectively.

The different matrix *A*_*C*_^*k*^ − *A*_*N*_^*k*^ of the differential PPI network between CPPIN and NPPIN in the *k*th cancer is defined as follows:

(8) Dk=[d11k…d1Mk⋮⋱⋮dM1k⋯dMMk]=[α^11,Ck−α^11,Nk…α^1M,Ck−α^1M,Nk⋮⋱⋮α^M1,Ck−α^M1,Nk⋯α^MM,Ck−α^MM,Nk],

where *k* = early and late stage bladder cancer; *d*_*ij*_^*k*^ denotes the protein association ability difference between CPPIN and NPPIN in the *k*th stage bladder cancer; and the matrix *D* ^*k*^ indicates the difference in network structure between CPPIN and NPPIN in the *k*th stage bladder cancer. In order to investigate carcinogenesis from the difference matrix *D* ^*k*^ between CPPIN and NPPIN of the *k*th stage bladder cancer in (8), a score, which we named the carcinogenesis relevance value (CRV), was presented to quantify the correlation of each protein in *D* ^*k*^ with the significance of carcinogenesis as follows [14]:

(9) $$\begin{array}{c} {\text{CRV}}^{k}=\left[\begin{array}{c} {\text{CRV}}_{1}^{k}\\ \vdots \\ {\text{CRV}}_{i}^{k}\\ \vdots \\ {\text{CRV}}_{M}^{k} \end{array}\right], \end{array}$$

where CRV_*i*_^*k*^ = ∑_*j*=1_^*M*^|*d*_*ij*_^*k*^|, and* k *= early and late stage bladder cancer.

The CRV_*i*_^*k*^ in (9) quantifies the differential extent of protein associations of the *i*th protein (the absolute sum of the *i*th row of *D* ^*k*^ in (8)) and the CRV^*k*^ can differentiate CPPIN from NPPIN in the *k*th stage bladder cancer. In other words, the CRV_*i*_^*k*^ in (9) could represent the network structure difference of the *i*th protein between the cancer and noncancer networks in the *k*th stage bladder cancer.

In order to investigate what proteins are more likely involved in the *k*th stage bladder cancer, we needed to calculate the corresponding empirical *P* value to determine the statistical significance of CRV_*i*_^*k*^. To determine the observed *P* value of each CRV_*i*_^*k*^, we repeatedly permuted the network structure of the candidate PPIN of the *k*th stage bladder cancer as a random network of the *k*th stage bladder cancer. Each protein in the random network of the *k*th stage bladder cancer will have its own CRV to generate a distribution of CRV_*i*_^*k*^ for *k* = early and late stage bladder cancer. Although there was random disarrangement of the network structure, the linkages of each protein were maintained. In other words, the proteins with which a particular protein interacted were permuted without changing the total number of protein interactions. This procedure was repeated 100,000 times and the corresponding *P* value was calculated as the fraction of random network structure in which the CRV_*i*_^*k*^ is at least as large as the CRV of the real network structure. According to the distributions of the CRV_*i*_^*k*^ of the random networks, the CRV_*i*_^*k*^ in (9) with a *P* value of less than or equal to 0.01 was regarded as a significant CRV and the corresponding protein was determined to be a significant protein in the carcinogenesis of the *k*th stage bladder cancer: a protein with a *P* value greater than 0.01 was removed from the list of significant proteins in carcinogenesis (in other words, if the *P* value of CRV_*i*_^*k*^ was greater than 0.01, then the *i*th protein was removed from the CRV_*i*_^*k*^ in (9) and the remainder in the CRV^*k*^ with *P* values of CRVs less than 0.01 were considered significant proteins of the *k*th stage bladder cancer).

Based on the *P* value of the CRVs for all proteins (*i* = 1,2,…, *M*) and the two stages of bladder cancer (*k* = early and late stage bladder cancer), we generated two lists of significant proteins for each of the two stages according to the CRV and the statistical assessment of each significant protein in CRV^*k*^ in (9). We found 152 significant proteins in early stage bladder cancer and 50 significant proteins in late stage bladder cancer. These proteins showed significant changes between the CPPIN and NPPIN in the carcinogenic process according to their corresponding stage of cancer and we suspected that these changes might play important roles in the carcinogenesis process of bladder cancer. These findings warrant further investigation.

The intersections of these significant proteins in the early and late stages of bladder cancer and their PPIs are known as the core network markers appearing in all stages of bladder cancer. In contrast, the unique significant proteins and their PPIs in each stage of bladder cancers are known as the specific network markers for each stage of cancer. We found that there were 18 significant proteins that could be classified as a core network marker in the whole carcinogenesis process of bladder cancer. We also found 134 significant proteins in the specific network marker of early stage bladder cancer and 32 significant proteins in the specific network marker of late stage bladder cancer.

### 2.5. Pathway Analysis

Much valuable cellular information can be found in the known pathways, which are useful for describing most “normal” biological phenomena. All of these known pathways are the result of repeated testing and verification and the entire pathway network has given definitions for most links. Therefore, the proteins we identified to be significant in the above network markers were mapped onto the known pathway networks (e.g., the KEGG or PANTHER pathway) to investigate significant pathways with the network marker and to explore the relationships between these pathways and the carcinogenesis of bladder cancer. This approach supports the view that systems biology can help identify significant network biomarkers in both normal and cancerous pathways to their roles in the pathogenesis of cancer.

Together with comprehensive pathway databases such as the Kyoto Encyclopedia of Genes and Genomes (KEGG), we used a series of bioinformatics pathway analysis tools to identify biologically relevant pathway networks [25]. KEGG includes manually curated biological pathways that cover three main categories: systems information (e.g., human diseases and drugs), genomics information (e.g., gene catalogs and sequence similarities), and chemical information (e.g., metabolites and biochemical reactions). At present, KEGG contains 134,511 distinct pathways generated from 391 original reference pathways [26]. Therefore, to investigate the pathways involved in carcinogenesis, the bioinformatics database DAVID [27, 28], which generates automatic outputs of the results from KEGG pathway analysis [27], was used for the pathway analysis of significant proteins identified in network markers to determine their roles in the pathogenesis of early and late stage bladder cancer. Our methodology does not contain the pathway analysis and gene set enrichment analysis. To complete our research results, we used the NOA software to do the pathway analysis and gene set enrichment analysis on biological processes, cellular components, and molecular functions [19, 29].

### 2.6. The Contribution of Protein Interaction Network Will Affect the Results of Biomarkers and the Evolution of Network Biomarkers

Our cancer PPI model is constructed from the differential expression of cancer and noncancer microarray data and data mining of PPI information from BioGRID database. So, the early and late stage bladder cancer CPPINs (cancer PPI networks) and NPPINs (noncancer PPI networks) are the results of our systems biology model using the original microarray data and PPI databases. There are three key factors that will affect the final results.

**Figure 2:**
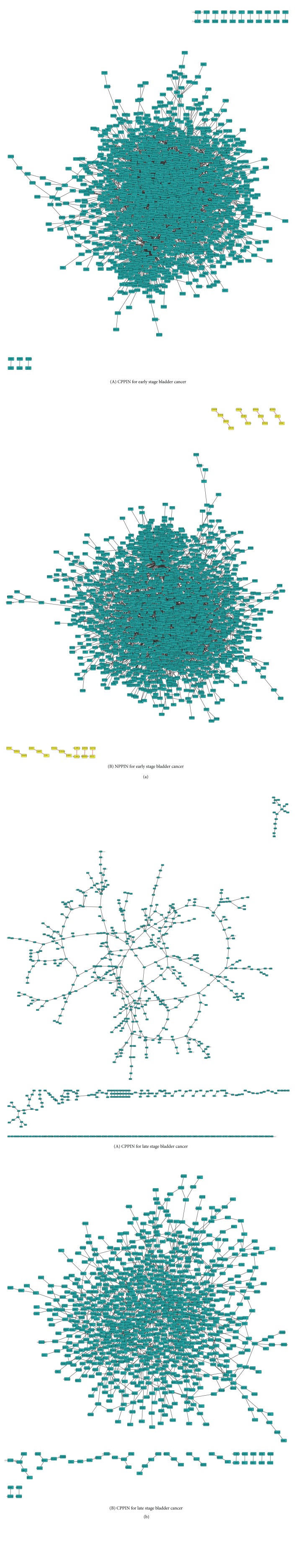
The constructed cancer PPIN (CPPIN) and noncancer PPIN (NPPIN) for early and late stage bladder cancer. The protein association numbers of CPPIN and NPPIN with respect to early and late bladder cancers are listed below (CPPIN/NPPIN): early stage bladder cancer (3388/3151) and late stage bladder cancer (634/1185). The figures are created using Cytoscape.

- The effect of different microarray data: we know that the microarray data has the shortage of irreproducible. That means even in the same case the microarray data does not promise to produce the same result as the previous ones. Also, for the same cancers, patients of different ethnics, different age, or different sex will give the different microarray data. This is the first factor to affect the final results.
- The effect of different original PPI databases: we know that PPI databases, such as BioGRID and MIPS, are constructed from putative and validated by wet-lab experiments. Due to the advances of many high-throughput experimental skills, the original PPI databases are evolved with time growing. The new updated original PPI databases are the second factor to affect the final results.
- The effect of systems biology model: microarray data, PPI databases, and PPI interaction model in (1) are employed to construct the PPI networks of normal and cancer cells by the maximum likelihood parameter estimation method (see Supplementary Material S.1). The AIC system order detection method (Supplementary Materials S.2) is employed to prune the false positive PPIs to obtain the real PPI networks of normal and cancer cells; that is, we use the so-called reverse engineering method to construct PPI networks of normal and cancer cells. Then the differential PPI network between cancer PPI network and normal PPI network is obtained in (8) to investigate PPI variations of each protein in the differential PPI network due to the carcinogenesis. Finally, the carcinogenesis value (CRV) based on PPI variations is also proposed to evaluate the significance of carcinogenesis for each protein of differential PPI network. Proteins with significant CRV (*P* value < 0.01) are considered as significant proteins of the cancer. The significant proteins in Table 3 are these significant proteins of early and late stage bladder cancers, and these proteins and their PPIs construct the interaction network in Figure 2. Finally, from the early to late stage bladder cancer network markers, we investigate the mechanism of carcinogenesis process with the help of databases (e.g., GO database, DAVID, and KEGG pathway database) and try to find multiple network target therapy of cancer. Unlike the conventional theoretical methods, which always give a single mathematical model for cancer network for a more detailed theoretical analysis, this study is to introduce a systems biology approach to cancer network markers based on real microarray data through the so-called reverse engineering, theoretical statistical method and data mining method in combination with big databases. These are the novelty and significance of our paper. Although we described the novelty of our systems biology model, we have validated our results by literature surveying in the research. In the future, our results will be validated by other researchers' wet-lab experiments, and we will modify our mathematical model again and again. This is the third key factor to affect the results. Although not directly, it will also have the influence on protein interaction network.

We also know that the biosystems are evolved with time. It is obvious that the early stage and late stage patients have very different symptoms; they are the key features for us to classify early and late stage bladder cancers. Since the two stage bladder cancer patients have great different symptoms, it is undoubted that the microarray data of these two stage patients will show to be quite different. As described above, the protein expression from microarray data is one of the key factors of our systems biology model to give the final CPPINs and NPPINs. And the CPPINs and NPPINs give the final network biomarkers from our systems biology model. So, the most important thing for the network biomarkers evolving is due to the evolution of microarray data at both stages of bladder cancer, which is inherent in the exhibition of cancer-related genes due to DNA mutations in the carcinogenesis process.

## 3. Results and Discussion

### 3.1. Time Evolution of the Network Biomarker from Early to Late Stage Bladder Cancer

In the first instance, we built the CPPIN and NPPIN for early and late stage bladder cancer (Figure 2). From the differential networks between CPPIN and NPPIN of early stage and late stage bladder cancer, we then calculated the CRV of each protein in the network structure. Screening in accordance with the *P* value of CRV, we determined the significant proteins of network markers for the two stages of bladder cancer. In the following, we will discuss the significant proteins identified in both stages and their intersection to reveal the carcinogenesis mechanisms from early to late stage bladder cancer.

### 3.2. Network Marker of Early and Late Stage Bladder Cancer

After *P* value (0.01) screening, we found that there were 152 and 50 significant proteins for early and late stage bladder cancer, respectively. In addition, their corresponding CRV values ranged between 4.1 and 158.5 and 3.4–29.9, respectively. These significant proteins and their PPIs were used to construct the network markers at early and late stage bladder cancer. The intersection network marker of both stages was a core feature that contained 18 significant proteins in carcinogenesis. We listed the 18 significant proteins and their corresponding CRV and *P* value in both stages of bladder cancer (Table 2). From this, we separately identified the 10 most significant proteins in early and late stage bladder cancer (Table 3). The full list of the 152 and 50 significant proteins for the two stages of bladder cancer is detailed in supplementary tables (Tables S1 and S2).

### 3.3. Pathway Analysis of Early Stage Bladder Cancer

**Figure 3:**
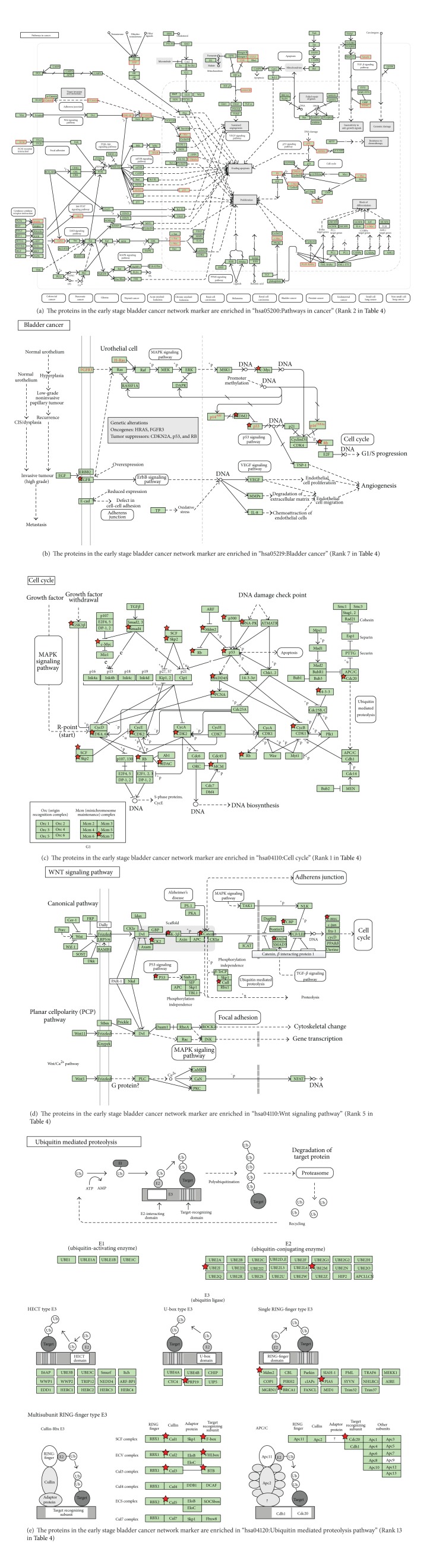
Overview of significant pathways in network marker of early stage bladder cancer. Among these KEGG pathways via DAVID tool (Table 4) showing a significant association with specific proteins of early stage bladder cancer, these molecular pathways are entitled with* P* value ≤ 0.05. It shows that these pathways are identified to play an important role in the carcinogenesis mechanism of early stage bladder cancer. The proteins in network markers of early stage bladder cancer highlighted by stars show potential targets in the pathways. Due to the different naming system, the same proteins in both these tables and in our text show the different names.

We analyzed the pathway of early stage bladder cancer using the DAVID database. Our initial observation revealed that several cancer pathways were hit by the 152 key proteins, including 11 genes in hsa05200: pathways in cancer (Figure 3(a)), 7 genes involved in prostate cancer, 6 genes involved in chronic myeloid leukemia, 5 genes involved in small cell lung cancer, 4 genes involved in bladder cancer, and 3 genes involved in thyroid cancer, respectively (Table 3). The four genes of hsa05219 involved in bladder cancer (TP53, MDM2, RN1, and MYC) are principal genes altered in urothelial carcinoma, which is highly related to metastatic bladder cancer and are significant targets of metastatic bladder cancer therapies [30] (Figure 3(b)). Thus, we now note that the 152 candidate proteins are not only related to bladder cancer, but also to other cancers and chronic myeloid leukemia. This would mean that common mechanisms exist between the development of the different cancers in the early stage of carcinogenesis.

Next, we proceeded to analyze the important pathways related to early stage bladder cancer (Table 4). Firstly, the cell cycle is composed of two consecutive periods (Figure 3(c)) characterized by DNA replication, sequential differentiation, and segregation of replicated chromosomes into two separate daughter cells. Both positive-acting and negative-acting proteins control the cells' entry and advancement through the cell cycle, which is composed of four distinct phases: G1 (Gap 1), S (synthesis), G2 (Gap 2), and M (mitosis) [31]. The G1 phase, where the cell grows in size, acts as a quality control check to determine whether the cell is ready to divide. The S phase is where the cell copies its DNA. The G2 phase involves cell checking as to whether all of its DNA has been correctly copied. The M phase is the cell division phase where the cell divides in two. Find out more about how cells prepare to divide and then share out their DNA and split in two. There are many reported discussions in regards to the cell cycle regulators and checkpoint functions involved in bladder cancer [32, 33]. Dysregulation of the cell cycle governs deviant cell proliferation in cancer. Losing the ability to control cell cycle checkpoints induces abnormal genetic instability. This may be due to the activation of tumorigenic mutations, which have been recognized in various tumors at different levels in the mitogenic signal transduction pathways: (1) ligands and receptors (receptor mutations of HER2/neu [ErB2] or the amplification of the HER2 gene), (2) downstream signal transduction networks (Raf/Ras/MAPK or PI3K-AKT-mTOR), and (3) regulatory genes of the cell cycle (cyclin D1/CDK4, CDK6, and cyclin E/CDK2) [34]. Increasing evidence convincingly implicates aberrant expression of cell cycle regulators in multiple cancers. Especially the restriction point (R) is the so-called G1 checkpoint. It separates the cell cycle into a mitogen-dependent phase and a growth factor-independent phase from the commitment to enter S phase. The G1 checkpoint commitment process integrates various and complex extracellular and intracellular signal transduction into the cell nucleus. Any malfunction of the G1 checkpoint may result in uncontrolled cell proliferation or genetic instability, possibly the origin of cancer or other diseases development [35].

The Wnt/*β*-catenin signaling pathways (Figure 3(d)) are composed of many functional networks, including a bundle of signaling pathways consisting of various proteins that transduce signals from the outside of a cell through the receptors on the cell surface and into the cell interior. They contribute significantly to the developmental process, particularly to direct cell attachment and proliferation. They are one of the most powerful signaling pathways and play critical roles in human development by controlling the genetic programs of embryonic development and adult homeostasis [36]. Under normal conditions, the Wnt signaling pathway is critical for healthy and normal development, while in adult cells, a dysregulated Wnt signaling pathway can lead to tumorigenesis. For this purpose, cancer cells must have the ability to switch from quiescent mode to proliferation mode, as well as switching between cell proliferation and cell invasion modes. Therefore, the Wnt signaling pathway participates in each of the stages of malignant cancer development and clearly contributes to human tumor progression. Much research has been reported on the relationship between Wnt signaling pathways and urological cancers (including bladder cancer) [37, 38].

**Figure 4:**
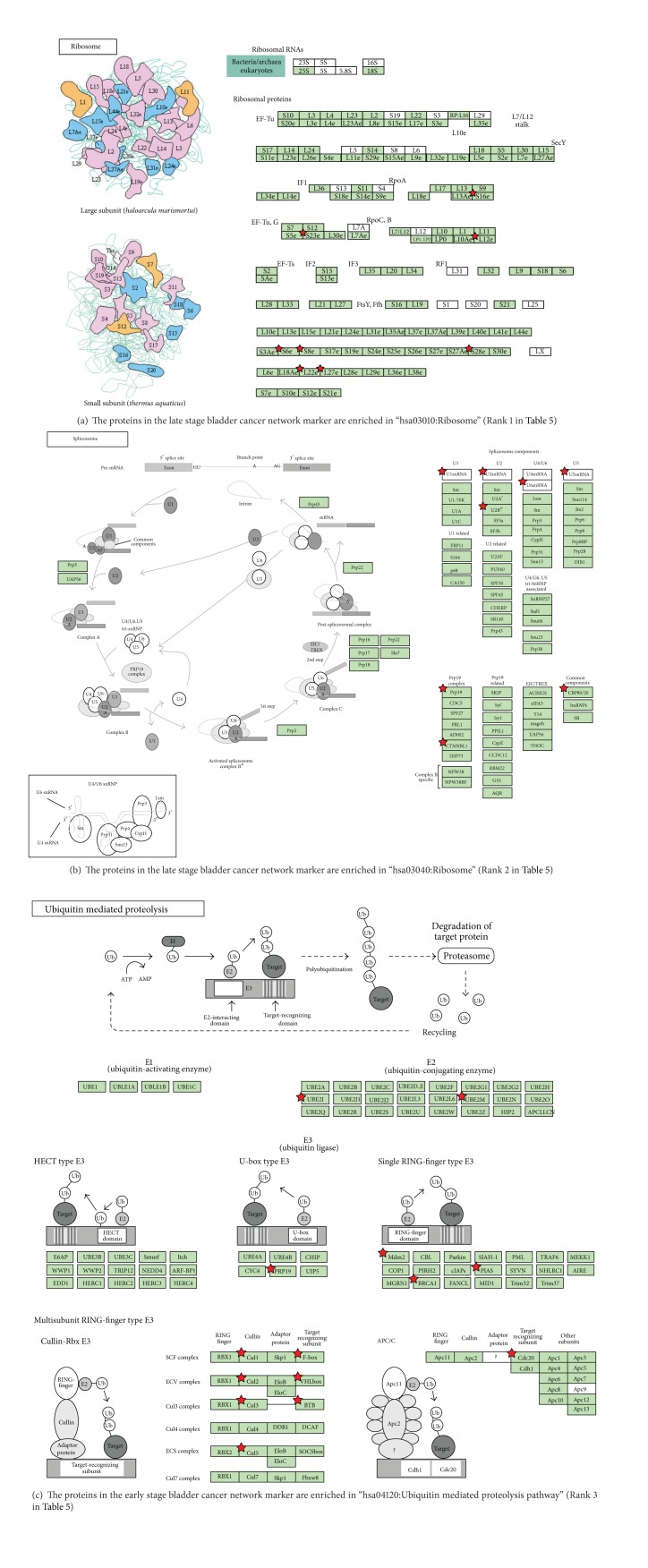
Overview of significant pathways in network marker of late stage bladder cancer. Among these KEGG pathways via DAVID tool (Table 5) showing a significant association with specific proteins of late stage bladder cancer, these molecular pathways are entitled with* P* value ≤ 0.05. It shows that these pathways are identified to play an important role in the carcinogenesis mechanism of late stage bladder cancer. The proteins in network markers of late stage bladder cancer highlighted by stars show potential targets in the pathways. Due to the different naming system, the same proteins in both these tables and in our text show the different names.

Other pathways identified in early stage bladder cancer, such as the Notch signaling pathway, adherens junctions, the TGF-*β* signaling pathway, ubiquitin-mediated proteolysis (Figures 3(e) and 4(c)), and the p53 signaling pathway are also associated with cancer [39–43].

The NOA analysis results of the pathway and gene enrichment analysis of the early stage bladder cancer is shown in Table 4(b): (1) Biological processes (2) Cellular components (3) Molecular functions. We saw that most of the biological processes are related to the metabolic processes. Second, about the cellular components, there are three of them related to the ribosome. Finally, about the molecular functions, there are RNA binding, heparin binding and cyclin binding, which are very different from the late stage bladder cancer.

### 3.4. Pathway Analysis of Late Stage Bladder Cancer

The most important results in this study as compared to our previous work are that we reveal related pathways of late stage bladder cancer in comparison to early stage cancer to reveal the evolution of network biomarkers in the carcinogenesis process. From Table 5, we observed that only three pathways, ribosome, spliceosome, and ubiquitin-mediated proteolysis pathways, were hit by the 50 candidate proteins identified in late stage bladder cancer. This is indicative of the evolution of cancer mechanisms from early stage bladder cancer.

The nucleolus is the site of ribosome biogenesis (Figure 4(a)). Due to the higher concentration of both RNA and proteins in the nucleolus than in the nucleoplasm, the nucleolus is easily detected by microscopy in living cells. From electron microscopy images, three major components were constantly exhibited by mammalian cells. They include fibrillar centers (FCs), which appear as surrounding structures of various sizes, with a very low electron opacity; the dense fibrillar component (DFC), which always constitutes a rim intimately accompanied with the fibrillar centers, composed of densely packed fibrils; and the granular component (GC), which is composed of granules that surround the fibrillar components. There is evidence that changes in nucleolar morphology and function may depend on both the rate and status of ribosome biogenesis and on the proliferative activity of cycling cells [44]. In cancer cells the upregulated ribosome biogenesis leads to an increased demand of ribosomal proteins for rRNA binding. In this way, after ribosome biogenesis alterations, cycling cells can activate the p53 pathway to ensure cell cycle arrest or alternatively to start the apoptotic program [45]. According to our analysis, there were eight significant proteins in the late stage cancer to hit the ribosome pathway.

Alternative splicing is a modification of the premessenger RNA (pre-mRNA) transcript in which internal noncoding regions of pre-mRNA (introns) are removed and then the remaining segments (exons) are joined (Figure 4(b)). The formation of mature messenger RNA (mRNA) is subsequently capped at its 5′ end and polyadenylated at its 3′ end, and transported out of the nucleus to be translated into protein in the cytoplasm. Most genes use alternative splicing to generate multiple spliced transcripts. These transcripts contain various combinations of exons resulting from different mRNA variants and then are synthesized as protein isoforms. The exons are always around 50–250 base pairs, whereas introns could be as long as several thousands of base pairs. For nuclear encoded genes, splicing takes place within the nucleus after or simultaneously with transcription. Splicing is necessary for the eukaryotic messenger RNA (mRNA) before it can be translated into a correct protein. The spliceosome is a dynamic intracellular macromolecular complex of multiple proteins and ribonucleoproteins (snRNPs). For many eukaryotic introns, the spliceosome carries out the two main functions of alternative splicing. First, it recognizes the intron-exon boundaries and second it catalyzes the cut-and-paste reactions that remove introns and concatenate exons. The various spliceosomal machinery complex is formed from 5 ribonucleo-protein (RNP) subunits, termed uridine-rich (U-rich) small nuclear RNP (snRNP), transiently associated with more than 760 non-snRNPs splicing factors (RNA helicases, SR splicing factors, etc.) [46, 47]. Each spliceosomal snRNP (U1, U2, U4, U5, and U6) consists of a uridine-rich small nuclear RNA (snRNA) complexed with a set of seven proteins known as canonical Sm core or SNRP proteins. The seven Sm proteins (B/B′, D1, D2, D3, E, F, and G) form a core ring structure that surrounds the RNA. All Sm proteins contain a conserved sequence motif in two segments (Sm1 and Sm2) that are responsible for the assembly and ordering of the snRNAs. They form the Sm core of the spliceosomal snRNPs [48] and process the pre-mRNA [49]. Spliceosomes not only catalyze splicing by a series of reactions, but they are also the main cellular machinery that guides splicing. Recently, scientists have found two natural compounds that can interfere with spliceosome function that also display anticancer activity in vitro and in vivo [50, 51]. Therefore, it is believable that inhibiting the spliceosome could act as a new target for anticancer drug development [52], and it should be validated in vivo or in vitro in the future.

The NOA analysis results of the pathway and gene enrichment analysis of the late stage bladder cancer is shown in Table 5(b): (1) Biological processes (2) Cellular components (3) Molecular functions. We saw most of the biological processes are related to cell cycle, which are different from the metabolic processes of early stage. Second, about the cellular components, there are complex evolution behaviors of the network compared with the early stage bladder cancer; there is only one intersection of these two stages that is ribonucleoprotein complex. It gives us many clues to develop evolutionary strategies for cancer target therapy. Finally, about the molecular functions, there are enzyme binding, protein binding and nucleotide binding, which are very different from the early stage bladder cancer. All the evolutionary behaviors from early to late stage bladder cancer let us reveal more hidden carcinogenesis mechanism.

### 3.5. Pathway Analysis of Both Early and Late Stage Bladder Cancer

The only pathway to intersect between early and late stage bladder cancer is the ubiquitin-mediated proteolysis pathway (Table 6). This means it is the only housekeeping pathway for bladder cancer and that the mechanisms of early and late stage bladder cancer are completely different. We hypothesize that this may be a novel concept for target therapy. Various other researches have never built a model in accordance with the network markers at the different stages of cancer. Our results show that the network markers of early stage hit common mechanisms and fundamental pathways, such as cell cycle, cell proliferation, and Wnt signaling, among others, which are implicated in various cancers. These provide clues in that early stage bladder cancer is active in many related pathways and we can assume that it is an active process to change the cell. In contrast, in the late stage of bladder cancer, the cells were inactive and close to silence. This may mean that the cells are close to death. Should we attempt to save these cells, we should aim to focus on the ribosome and spliceosome pathways. Of course ubiquitin-mediated proteolysis pathways are both active in early and late stage cancer.

## 4. Conclusions

Bladder cancer is among the 10 most common forms of carcinoma in the USA and worldwide. It is a lethal disease like other cancers and understanding the carcinogenesis mechanism can help to develop new therapeutic strategy. Identifying the PPI interface to develop small molecule inhibitors has become a new direction for targeted cancer therapy. This study, which follows from our prior work, analyzes the carcinogenesis mechanism from early to late stage bladder cancer using a network-based biomarker evolution approach. Other research studies do not distinguish network markers between these two stages of bladder cancer. Thus, our approach is advantageous in that it can provide added insight into the significant network marker evolution of the carcinogenesis process of bladder cancer. The network markers and their related pathways identified in early stage bladder cancer are mostly related to ordinary cancer mechanisms, which just show a highly active state of the early stage and cannot reveal additional novel results. All of these results should be validated in vivo or in vitro in the future. However, from the two specific and significant pathways identified in late stage bladder cancer, ribosome pathway and spliceosome pathway, we identified a novel result, which has potential to become a target for cancer therapy. The only core pathway in these two stages is the ubiquitin-mediated proteolysis pathway, which is a significant cue of carcinogenesis from early to late stage bladder cancer. Applying our method to study more cancers and more classification groups (such as stage, age, ethics, and sex) will give us further insight into the various pathogenesis mechanisms.

## Supplementary Material

There are two parts of the supplementary materials. The first one: S.1 is the Parameter Identification of Regression Model in Equation (1) by Maximum Likelihood Method; and the second part: S.2 is the Determination of significant protein associations by AIC and Student's t-test.

## Figures and Tables

**Table 1:** Descriptive information on datasets extracted from the GEO database used in this study.

Cancer	GEO accession number	Early stage	Late stage	Adjacent normal	Platform
Bladder cancer	GSE13507	106	37	58	GPL6102

Cases are grouped by cancer and surrounding normal tissues came from human patients of early stage and late bladder cancer.

**Table 2:** The 18 identified significant proteins of core network marker in both early and late stage bladder cancers.

Common network marker of early and late stage bladder cancer				
Protein	CRV-early	*P* value-early	CRV-late	*P* value-late
UBC	29.91709	<1*e − *5	158.5321	<1*e − *5
CUL3	27.96694	<1*e − *5	13.0117	<1*e − *5
CUL5	14.97713	<1*e − *5	4.834916	0.002872
RPL22	10.47367	<1*e − *5	8.110447	<1*e − *5
SUMO2	8.391421	<1*e − *5	10.34113	<1*e − *5
APP	6.933807	<1*e − *5	11.47363	<1*e − *5
SH3KBP1	6.765387	<1*e − *5	4.447911	0.00619
PTBP1	6.740458	<1*e − *5	4.511016	0.005506
ELAVL1	6.635085	<1*e − *5	10.77056	<1*e − *5
SIRT7	5.55441	3.13*E − *05	7.656515	<1*e − *5
MYC	4.6109	0.00072	13.0423	<1*e − *5
HSP90AA1	4.60136	0.00072	6.513345	0.000123
COPS5	3.898548	0.003163	6.601779	9.22*E − *05
ESR1	3.873735	0.003383	5.6189	0.000614
BRCA1	3.788256	0.00415	11.5863	<1*e − *5
TERF2IP	3.680287	0.00487	5.202998	0.00149
SOX2	3.534024	0.007219	5.495338	0.00076
CUL1	3.521039	0.00736	13.2669	<1*e − *5

**Table 3:** The identified top 20 significant proteins in both early and late stage bladder cancer individually.

Early stage bladder cancer (*N* = 107)			Late stage bladder cancer (*N* = 107)		
CRV	Name	*P* value	CRV	Name	*P* value
UBC	29.91709	<1*e − *5	UBC	158.5321	<1*e − *5
CUL3	27.96694	<1*e − *5	VCAM1	20.98798103	<1*e − *5
RIOK2	16.02326	<1*e − *5	RPS13	20.09693015	<1*e − *5
CUL5	14.97713	<1*e − *5	TP53	19.5883	<1*e − *5
RPS23	12.13218	<1*e − *5	HDAC1	19.2879	<1*e − *5
RPL12	10.87102	<1*e − *5	HSPA8	17.24137906	<1*e − *5
RPL22	10.47367	<1*e − *5	RPS27A	17.23738059	<1*e − *5
RANBP2	9.8086	<1*e − *5	TUBB	17.03734405	<1*e − *5
PAN2	9.521207	<1*e − *5	CDK2	16.7366	<1*e − *5
DHX9	9.47832	<1*e − *5	VIM	15.89214155	<1*e − *5
RPS8	8.722495	<1*e − *5	KIAA0101	15.8188	<1*e − *5
RPL27	8.641642	<1*e − *5	ITGA4	15.69058519	<1*e − *5
SUMO2	8.391421	<1*e − *5	GSK3B	15.44597966	<1*e − *5
HNRNPH3	8.011681	<1*e − *5	EEF1A1	14.21690842	<1*e − *5
CDC5L	7.950851	<1*e − *5	RUVBL2	13.63207486	<1*e − *5
RUVBL1	7.887244	<1*e − *5	PCNA	13.3217	<1*e − *5
SF3A1	7.468209	<1*e − *5	CUL1	13.2669	<1*e − *5
APP	6.933807	<1*e − *5	MYC	13.0423	<1*e − *5
CCT3	6.860228	<1*e − *5	CUL3	13.0117	<1*e − *5
SH3KBP1	6.765387	<1*e − *5	HNRNPA0	12.15264603	<1*e − *5

**(a):** 

Rank	Term	Count	Symbol	*P* value
1	hsa04110:Cell cycle	13	YWHAZ, CREBBP, TP53, PRKDC, RB1, CDK2, HDAC2, EP300, HDAC1, PCNA, MDM2, MYC, andCUL1	1.50*E − *14
2	hsa05200:Pathways in cancer	11	TRAF2, EP300, HDAC2, HDAC1, CREBBP, TP53, MDM2, RB1, MYC, CDK2, and CTNNB1	3.51*E − *07
3	hsa05215:Prostate cancer	7	EP300, CREBBP, TP53, MDM2, RB1, CDK2, and CTNNB1	1.45*E − *06
4	hsa05220:Chronic myeloid leukemia	6	HDAC2, HDAC1, TP53, MDM2, RB1, and MYC	1.32*E − *05
5	hsa04310:Wnt signaling pathway	6	EP300, CREBBP, TP53, MYC, CUL1, and CTNNB1	3.78*E − *04
6	hsa05222:Small cell lung cancer	5	TRAF2, TP53, RB1, MYC, and CDK2	4.04*E − *04
7	hsa05219:Bladder cancer	4	TP53, MDM2, RB1, and MYC	7.24*E − *04
8	hsa04330:Notch signaling pathway	4	EP300, HDAC2, HDAC1, and CREBBP	0.001008
9	hsa04520:Adherens junction	4	EP300, CREBBP, SRC, and CTNNB1	0.00418
10	hsa04350:TGF-beta signaling pathway	4	EP300, CREBBP, MYC, and CUL1	0.005889
11	hsa05016:Huntington's disease	5	EP300, HDAC2, HDAC1, CREBBP, and TP53	0.006736
12	hsa05216:Thyroid cancer	3	TP53, MYC, and CTNNB1	0.00676
13	hsa04120:Ubiquitin mediated proteolysis	4	CUL3, MDM2, BRCA1, and CUL1	0.020254
14	hsa05213:Endometrial cancer	3	TP53, MYC, and CTNNB1	0.020793
15	hsa05214:Glioma	3	TP53, MDM2, and RB1	0.029762
16	hsa04115:p53 signaling pathway	3	TP53, MDM2, and CDK2	0.034267
17	hsa05218:Melanoma	3	TP53, MDM2, and RB1	0.037091
18	**hsa05210:Colorectal cancer**	**3**	**TP53, MYC, and CTNNB1**	**0.050311**
19	**hsa04210:Apoptosis**	**3**	**TRAF2, IRAK1, and TP53**	**0.053574**
20	**hsa03450:Non-homologous end-joining**	**2**	**XRCC6and PRKDC**	**0.05487**
21	**hsa04916:Melanogenesis**	**3**	**EP300, CREBBP, and CTNNB1**	**0.067353**
22	**hsa04114:Oocyte meiosis**	**3**	**YWHAZ, CDK2, and CUL1**	**0.080913**
23	**hsa04722:Neurotrophin signaling pathway**	**3**	**IRAK1, YWHAZ, and TP53**	**0.099295**

The significant pathways via DAVID Bioinformatics database are selected for the 152 significant proteins in carcinogenesis. Black background indicates *P* value > 0.05.

**(b):** 

GO:term	*P* value	Corrected *P* value	*R*	*T*	*G*	*O*	Term name
(1) Biological processes							
0044260	5.3*E − *11	1.7*E − *8	14791	19	3428	18	Cellular macromolecule metabolic process
0043170	7.3*E − *10	2.4*E − *7	14791	19	3975	18	Macromolecule metabolic process
0044237	3.6*E − *8	1.2*E − *5	14791	19	4963	18	Cellular metabolic process
0006414	1.4*E − *7	4.7*E − *5	14791	19	101	5	Translational elongation
0019538	1.0*E − *6	3.2*E − *4	14791	19	2528	13	Protein metabolic process
0008152	1.1*E − *6	3.6*E − *4	14791	19	6033	18	Metabolic process
0044238	1.7*E − *6	5.6*E − *4	14791	19	5258	17	Primary metabolic process
0016071	4.4*E − *6	0.0014	14791	19	364	6	mRNA metabolic process
0044267	3.3*E − *5	0.0110	14791	19	1883	10	Cellular protein metabolic process
0009987	1.2*E − *4	0.0406	14791	19	9216	19	Cellular process

(2) Cellular components							
0030529	7.9*E − *10	7.8*E − *8	16768	18	510	9	Ribonucleoprotein complex
0032991	1.8*E − *7	1.8*E − *5	16768	18	3312	14	Macromolecular complex
0043228	1.2*E − *6	1.2*E − *4	16768	18	2051	11	Non-membrane-bounded organelle
0043232	1.2*E − *6	1.2*E − *4	16768	18	2051	11	Intracellular non-membrane-bounded organelle
0005840	1.5*E − *6	1.5*E − *4	16768	18	196	5	Ribosome
0005829	2.0*E − *6	2.0*E − *4	16768	18	1269	9	Cytosol
0043229	8.3*E − *6	8.2*E − *4	16768	18	8759	18	Intracellular organelle
0043226	8.5*E − *6	8.4*E − *4	16768	18	8773	18	Organelle
0044445	1.7*E − *5	0.0016	16768	18	150	4	Cytosolic part
0033279	2.8*E − *4	0.0287	16768	18	123	3	Ribosomal subunit

(3) Molecular functions							
0003735	1.0*E − *6	1.1*E − *4	15767	19	161	5	Structural constituent of ribosome
0003723	1.8*E − *4	0.0193	15767	19	755	6	RNA binding
0005198	8.0*E − *4	0.0825	15767	19	643	5	Structural molecule activity
0031625	0.0013	0.1360	15767	19	45	2	Ubiquitin protein ligase binding
0003678	0.0013	0.1360	15767	19	45	2	DNA helicase activity
0004535	0.0024	0.2480	15767	19	2	1	Poly(A)-specific ribonuclease activity
0033130	0.0048	0.4956	15767	19	4	1	Acetylcholine receptor binding
0008201	0.0079	0.8140	15767	19	112	2	Heparin binding
0030332	0.0096	0.9890	15767	19	8	1	Cyclin binding
0004386	0.0129	1	15767	19	145	2	Helicase activity

*R*: number of genes in reference set.

*T*: number of genes in test set.

*G*: number of genes annotated by given term in reference set.

*O*: number of genes annotated by given term in test set.

**(a):** 

Rank	Term	Count	Symbol	*P* value
1	hsa03010:Ribosome	8	RPS28, RPS16, RPL22, RPL27, RPL12, RPS6, RPS8, and RPS23	2.26*E − *07
2	hsa03040:Spliceosome	5	HSPA1L, CDC5L, SF3A1, SNRPE, and HNRNPU	0.004054716
3	hsa04120:Ubiquitin mediated proteolysis	4	CUL3, CUL5, BRCA1, and CUL1	0.034906958

The significant pathways via DAVID Bioinformatics database are selected for the 50 significant proteins in carcinogenesis.

**(b):** 

GO:term	*P* value	Corrected *P* value	*R*	*T*	*G*	*O*	Term name
(1) Biological processes							
GO:0045786	1.8*E − *6	0.0010	14791	18	178	5	Negative regulation of cell cycle
GO:0022402	2.4*E − *6	0.0014	14791	18	562	7	Cell cycle process
GO:0007050	8.4*E − *6	0.0049	14791	18	111	4	Cell cycle arrest
GO:0051726	8.5*E − *6	0.0049	14791	18	435	6	Regulation of cell cycle
GO:0060710	1.3*E − *5	0.0080	14791	18	5	2	Chorioallantoic fusion
GO:0044260	1.4*E − *5	0.0081	14791	18	3428	13	Cellular macromolecule metabolic process
GO:0051052	1.5*E − *5	0.0091	14791	18	130	4	Regulation of DNA Metabolic process
GO:0008629	2.6*E − *5	0.0155	14791	18	49	3	Induction of apoptosis by intracellular signals
GO:0006917	2.8*E − *5	0.0162	14791	18	313	5	Induction of apoptosis
GO:0012502	2.8*E − *5	0.0165	14791	18	314	5	Induction of programmed cell death

(2) Cellular components							
GO:0032991	5.2*E − *10	5.5*E − *8	16768	18	3312	16	Macromolecular complex
GO:0005829	1.4*E − *7	1.5*E − *5	16768	18	1269	10	Cytosol
GO:0043234	2.5*E − *6	2.6*E − *4	16768	18	2748	12	Protein complex
GO:0005654	6.1*E − *6	6.4*E − *4	16768	18	465	6	Nucleoplasm
GO:0044428	6.4*E − *5	0.0067	16768	18	1932	9	Nuclear part
GO:0000307	9.8*E − *5	0.0102	16768	18	14	2	Cyclin-dependent protein kinase holoenzyme complex
GO:0030529	1.5*E − *4	0.0163	16768	18	510	5	Ribonucleoprotein complex
GO:0031461	4.9*E − *4	0.0516	16768	18	31	2	Cullin-RING ubiquitin ligase complex
GO:0022627	7.4*E − *4	0.0777	16768	18	38	2	Cytosolic small ribosomal subunit
GO:0043626	0.0010	0.1116	16768	18	1	1	PCNA complex

(3) Molecular functions							
GO:0019899	3.1*E − *5	0.0037	15767	18	584	6	Enzyme binding
GO:0005515	1.1*E − *4	0.0129	15767	18	8097	17	Protein binding
GO:0030337	0.0011	0.1335	15767	18	1	1	DNA polymerase processivity factor activity
GO:0000701	0.0011	0.1335	15767	18	1	1	Purine-specific mismatch base pair DNA N-glycosylase activity
GO:0031625	0.0011	0.1384	15767	18	45	2	Ubiquitin protein ligase binding
GO:0000166	0.0021	0.2510	15767	18	2283	8	Nucleotide binding
GO:0035033	0.0022	0.2669	15767	18	2	1	Histone deacetylase regulator activity
GO:0004696	0.0022	0.2669	15767	18	2	1	Glycogen synthase kinase 3 activity
GO:0000700	0.0022	0.2669	15767	18	2	1	Mismatch base pair DNA N-glycosylase activity
GO:0005200	0.0031	0.3705	15767	18	74	2	Structural constituent of cytoskeleton

*R*: number of genes in reference set.

*T*: number of genes in test set.

*G*: number of genes annotated by given term in reference set.

*O*: number of genes annotated by given term in test set.

**Table 6:** The pathways analysis for 18 significant proteins in early and late stage bladder cancer carcinogenesis.

Rank	Term	Count	Symbol	*P* value
1	hsa03010:Ribosome	4	CUL3, CUL5, BRCA1, and CUL1	1.4*E − *3
