# Supplementary material for: Evolution of Network Biomarkers from Early to Late Stage Bladder Cancer Samples
Source: Biomed Res Int. 2014 Sep 18;2014:159078. doi: 10.1155/2014/159078 (PMC4189772; doi:10.1155/2014/159078)
Supplement: Supplementary file 1 — There are two parts of the supplementary materials. The first one: S.1 is the Parameter Identification of Regression Model in Equation (1) by Maximum Likelihood Method; and the second part: S.2 is the Determination of significant protein associations by AIC and Student's t-test. [file 159078.f1.pdf]

Supplementary Materials

S.1 Parameter Identification of Regression Model in Equation (1) by Maximum Likelihood Method

Equation (1) can be written as the following requiring form

$$x_i[n] = \left[x_1[n] \ \cdots \ x_{Mi}[n]\right]\begin{bmatrix}\alpha_{i1} \\ \vdots \\ \alpha_{iM_i}\end{bmatrix} + \omega_i[n] = \phi_i[n]\cdot\theta_i + \omega_i[n] \qquad \text{(S1)}$$

where $\phi_i[n]$ denotes the regression vector which can be obtained from microarray data, θ_i is the parameter vector to be estimated. Suppose that there are *m* samples, then it is easy to acquired values of $\left\{x_i[n] \ \phi_i[n]\right\}$ for $n \in \left\{1, \ \ldots \ ,m\right\}$. In this case, equation (2) for different samples can be represented as the following vector form.

$$\begin{bmatrix}x_i[1] \\ \vdots \\ x_i[m]\end{bmatrix} = \begin{bmatrix}\phi_i[1] \\ \vdots \\ \phi_i[m]\end{bmatrix}\cdot\theta_i + \begin{bmatrix}\omega_i[1] \\ \vdots \\ \omega_i[m]\end{bmatrix} \qquad \text{(S2)}$$

where $\phi_i[m] = [x_i(m)\cdots x_{Mi}(n)], \theta_i = [\alpha_{i1}\cdots\alpha_{iM_i}]$

For simplicity, it can be represented as follows.

$$X_i = \Phi_i\cdot\theta_i + e_i \qquad \text{(S3)}$$

where $e_i = [w_1(1)\cdots w_i(m)]^T$.

In equation (A3), the noise e_i for different samples was regarded as independent random variables of normal distribution with zero mean and unknown variance σ_i^2, i.e., $E\left\{e_i\right\} = 0$, and $\Sigma_i = E\left\{e_i e_i^T\right\} = \sigma_i^2 I$, where *I* is the identity matrix. The probability density function of e_i is given as follows.

$$p(e_i) = \frac{1}{((2\pi)^m \det\Sigma_i)^{1/2}}\exp(-\frac{1}{2}e_i^T\,\Sigma_i^{-1}\,e_i) \qquad \text{(S4)}$$

From equation (A4), we can obtain the likelihood function

$$L(\theta_i,\sigma_i^2)=p(\theta_i,\sigma_i^2)=\frac{1}{(2\pi\sigma_i^2)^{m/2}}\exp(-\frac{(X_i-\Phi_i\theta_i)^T(X_i-\Phi_i\theta_i)}{2\sigma_i^2}) \quad \text{(S5)}$$

Maximum likelihood estimation method aims at finding θ_i and σ_i^2 to maximize the likelihood function in equation (A5). In order to simplify the computation, it is practical to take the logarithm of the likelihood function, which yields the following log-likelihood function:

$$\log L(\theta_i,\sigma_i^2)=-\frac{m}{2}\log(2\pi\sigma_i^2)-\frac{1}{2\sigma_i^2}\sum_{n=1}^{m}\left[y_i[n]-\phi_i[n]\cdot\theta_i\right]^2 \quad \text{(S6)}$$

where $x_i[n]$ and $\phi_i[n]$ are the *n*-th element of X_i and Φ_i in (A3), respectively.

By the maximum likelihood parameter estimation method, we expect the log-likelihood function to have the maximum at $\theta_i=\hat{\theta}_i$ and $\sigma_i^2=\hat{\sigma}_i^2$. The necessary conditions for the maximum likelihood estimates $\hat{\theta}_i$ and $\hat{\sigma}_i^2$ must conform to the following two equations.

$$\begin{aligned}&\frac{\partial \log L(\theta_i,\sigma_i^2)}{\partial\theta_i}=0\\&\frac{\partial \log L(\theta_i,\sigma_i^2)}{\partial\sigma_i^2}=0\end{aligned} \quad \text{(S7)}$$

The estimated parameters $\hat{\theta}_i$ and $\hat{\sigma}_i^2$ are shown below,

$$\hat{\theta}_i=(\Phi_i^T\Phi_i)^{-1}\Phi_i^TY_i \quad \text{(S8)}$$

$$\hat{\sigma}_i^2=\frac{1}{m}\sum_{n=1}^{m}\left[x_i[n]-\phi_i[n]\cdot\hat{\theta}_i\right]^2=\frac{1}{m}(X_i-\Phi_i\cdot\hat{\theta}_i)^T(X_i-\Phi_i\cdot\hat{\theta}_i) \quad \text{(S9)}$$

where X_i and Φ_i can be obtained from the microarray in the rough PPIN. Since there are two data sets of microarray data, two association parameters for cancer and non-cancer were separately identified.

S.2 Determination of significant protein associations by AIC and Student's t-test

When association parameters of all the proteins in rough PPIN were identified as equation (2), significant protein associations were determined by parameter estimates $\hat{\alpha}_{ij}$ of their association abilities. In order to determine whether the association was significant or not, Akaike Information Criterion (AIC) and Student's t-test, which is used to calculate the *p*-values of the association abilities, are employed to detect the system model order (or the number of model parameters) and determine the significance of our model parameters. The AIC, a method for model order detection, attempts to include both the estimated residual variance and model complexity in one statistic. AIC decreases as residual variance decreases, and increases as the number of parameters increases. As the expected residual variance decreases with increasing parameter numbers for excessive model complexity, a minimum should appear near the correct parameter number. Thus, the AIC criterion, in which estimated parameters were obtained above, was used to select model structure. Due to computation efficiency, it is impractical to compute the AIC statistics for all possible regression models. Here, the stepwise regression method which combines forward selection method and backward elimination method was applied to compute the AIC statistics. Once the estimated association parameters were examined using the AIC model detection criteria, the student's t-test was employed to calculate the *p*-values for the association abilities under the null hypothesis $H_0 : \alpha_{ij} = 0$ to determine the significant protein associations. The *p*-values computed were then adjusted by Bonferroni correction to avoid a lot of spurious positives. The associations which adjusted *p*-value ≤ 0.05 were determined as significant associations and were preserved in the protein association network [1].

Briefly, we use the AIC method to obtain how many system orders, which mean the numbers of interactions, in the dynamic system of the association abilities (model). We use the above maximum likelihood estimate method to identify the parameter $\hat{\alpha}_{ij}$ and then employ AIC and student t-test to

calculate *p*-values of association abilities for determining the significant PPIs for the target protein *i* by pruning the insignificant PPIs.

Table S3 - The identified significant proteins in early stage bladder cancer.

Early Stage Bladder cancer (N=107)					
**CRV**	**Name**	***p*-value**	**CRV**	**name**	***p*-value**
UBC	158.5321	< 1e-5	ELAVL1	10.77056	< 1e-5
VCAM1	20.98798	< 1e-5	DDX3X	10.54198	< 1e-5
RPS13	20.09693	< 1e-5	FLNA	10.46666	< 1e-5
TP53	19.5883	< 1e-5	U2AF2	10.44709	< 1e-5
HDAC1	19.2879	< 1e-5	RPS4X	10.39146	< 1e-5
HSPA8	17.24138	< 1e-5	SUMO2	10.34113	< 1e-5
RPS27A	17.23738	< 1e-5	RPL17	10.2199	< 1e-5
TUBB	17.03734	< 1e-5	SNCA	9.948332	< 1e-5
CDK2	16.7366	< 1e-5	RPL15	9.770824	< 1e-5
VIM	15.89214	< 1e-5	YWHAQ	9.58211	< 1e-5
KIAA0101	15.8188	< 1e-5	SF3A2	9.370873	< 1e-5
ITGA4	15.69059	< 1e-5	GAPDH	9.320667	< 1e-5
GSK3B	15.44598	< 1e-5	TRAF2	9.2003	< 1e-5
EEF1A1	14.21691	< 1e-5	KDM1A	9.161272	< 1e-5
RUVBL2	13.63207	< 1e-5	COPS4	9.159837	< 1e-5
PCNA	13.3217	< 1e-5	BAG3	9.079839	< 1e-5
CUL1	13.2669	< 1e-5	UBQLN4	8.89109	< 1e-5
MYC	13.0423	< 1e-5	SRC	8.8026	< 1e-5
CUL3	13.0117	< 1e-5	YWHAZ	8.7995	< 1e-5
HNRNPA0	12.15265	< 1e-5	IQGAP1	8.567994	< 1e-5
EP300	12.1078	< 1e-5	TSC22D1	8.329745	< 1e-5
CREBBP	12.0995	< 1e-5	RPL22	8.110447	< 1e-5
BRCA1	11.5863	< 1e-5	CTNNB1	7.6796	< 1e-5
APP	11.47363	< 1e-5	SIRT7	7.656515	< 1e-5
SMAD4	11.11657	< 1e-5	SF3B3	7.545664	0.00001
HDAC2	11.0938	< 1e-5	PRPF19	7.543978	0.00001
RPS15A	11.07202	< 1e-5	AURKA	7.400529	0.00002
EFTUD2	11.01847	< 1e-5	CDK8	7.126101	0.00002
ATXN1	10.93067	< 1e-5	PA2G4	7.115277	0.00002
SRSF1	10.82556	< 1e-5	TK1	6.94417	0.00005

Early Stage Bladder cancer (cont.)					
**CRV**	**Name**	***p*-value**	**CRV**	**Name**	***p*-value**
RB1	6.8922	0.00006	LRRK2	5.79669	0.00044
EIF3L	6.855981	0.00006	EGFR	5.665875	0.00054
NR3C1	6.759946	0.00008	SUV39H1	5.653659	0.00057
RPS12	6.636237	0.00008	NR4A1	5.622039	0.00061
PRNP	6.61903	0.00009	ESR1	5.6189	0.00061
COPS5	6.601779	0.00009	WAS	5.57976	0.00070
CAND1	6.563826	0.00010	THRA	5.542012	0.00074
SMARCC2	6.544437	0.00011	SOX2	5.495338	0.00076
HSP90AA1	6.513345	0.00012	TERF1	5.4642	0.00082
GABARAPL2	6.507613	0.00012	HDAC8	5.443446	0.00087
HGS	6.468401	0.00013	XRCC6	5.2871	0.00127
ZBTB16	6.461729	0.00014	CEBPB	5.2303	0.00142
ICT1	6.415951	0.00015	TERF2IP	5.202998	0.00149
SMARCA4	6.38881	0.00016	PAFAH1B1	5.178814	0.00154
KAT2B	6.359971	0.00016	FOS	5.109807	0.00173
ENO1	6.357504	0.00017	PIAS4	5.087386	0.00180
CAV1	6.342663	0.00018	JAK1	5.038004	0.00194
WIBG	6.263279	0.00022	UBE2M	5.031314	0.00194
DAZAP2	6.223404	0.00024	TUBB6	5.010131	0.00201
PRKAR1A	6.172757	0.00025	LIG4	4.977367	0.00217
ARRB1	6.108699	0.00028	ITK	4.930477	0.00237
MCM7	6.101756	0.00028	CDK6	4.921106	0.00241
EIF1B	6.022725	0.00031	TOP2A	4.896717	0.00255
FN1	6.009818	0.00031	CHAF1A	4.889059	0.00257
MTA2	5.969311	0.00034	CUL2	4.861326	0.00272
SKP2	5.948008	0.00035	TPM1	4.849335	0.00279
JAK2	5.908504	0.00037	AURKB	4.848723	0.00281
CBX3	5.900383	0.00038	GRB2	4.847256	0.00282
CCNB1	5.899322	0.00038	CUL5	4.834916	0.00287
SF3B4	5.898706	0.00038	XPO5	4.83468	0.00288
MEN1	5.869402	0.00040	PPP1CB	4.823871	0.00293
HDAC4	5.8659	0.00040	RANGAP1	4.812317	0.00300
KHSRP	5.861757	0.00040	VHL	4.745417	0.00337
PSMA3	5.8022	0.00044	UBE2I	4.714676	0.00367
Dlg4	5.799353	0.00044	EIF6	4.6331	0.00433

Early Stage Bladder cancer (cont.)		
**CRV**	**Name**	***p*-value**
NEDD8	4.595795	0.00468
GADD45A	4.577573	0.00487
CSNK2A1	4.574648	0.00488
MDM2	4.5647	0.00503
UBD	4.562146	0.00507
KEAP1	4.51156	0.00551
PTBP1	4.511016	0.00551
ISG15	4.4856	0.00578
SH3KBP1	4.447911	0.00619
CDC20	4.382527	0.00711
RELA	4.378365	0.00721
SLC25A4	4.36766	0.00735
PRDX3	4.360177	0.00747
MDC1	4.328266	0.00788
MDFI	4.327818	0.00788
ITSN1	4.320207	0.00803
PPP1CA	4.294617	0.00833
P4HB	4.263702	0.00882
SIRT1	4.241573	0.00911
CIRBP	4.22074	0.00938
IRAK1	4.1157	0.01073
PRKDC	4.0781	0.01122

Table S3 - The identified significant proteins in late stage bladder cancer

Late Stage Bladder cancer (N=58)					
**CRV**	**Name**	***p*-value**	**CRV**	**name**	***p*-value**
UBC	29.91709	< 1e-5	MYC	4.6109	0.00072
CUL3	27.96694	< 1e-5	HSP90AA1	4.60136	0.00072
RIOK2	16.02326	< 1e-5	HSPA1L	4.399888	0.00094
CUL5	14.97713	< 1e-5	RPS28	4.340192	0.001112
RPS23	12.13218	< 1e-5	SRSF11	3.9789	0.002662
RPL12	10.87102	< 1e-5	COPS5	3.898548	0.003163
RPL22	10.47367	< 1e-5	ESR1	3.873735	0.003383
RANBP2	9.8086	< 1e-5	POLD1	3.791715	0.004134
PAN2	9.521207	< 1e-5	BRCA1	3.788256	0.00415
DHX9	9.47832	< 1e-5	ILK	3.700961	0.004745
RPS8	8.722495	< 1e-5	RPS16	3.681108	0.00487
RPL27	8.641642	< 1e-5	TERF2IP	3.680287	0.00487
SUMO2	8.391421	< 1e-5	KHDRBS1	3.678307	0.004886
HNRNPH3	8.011681	< 1e-5	IQCB1	3.667747	0.005136
CDC5L	7.950851	< 1e-5	SNRPE	3.629871	0.005638
RUVBL1	7.887244	< 1e-5	UBASH3B	3.563628	0.006577
SF3A1	7.468209	< 1e-5	SOX2	3.534024	0.007219
APP	6.933807	< 1e-5	MAP3K11	3.524654	0.00736
CCT3	6.860228	< 1e-5	CUL1	3.521039	0.00736
SH3KBP1	6.765387	< 1e-5	MRPS22	3.436393	0.008675
PTBP1	6.740458	< 1e-5			
ELAVL1	6.635085	< 1e-5			
STAU1	6.141361	1.57E-05			
RBBP4	5.797126	1.57E-05			
SIRT7	5.55441	3.13E-05			
USP9X	5.527938	3.13E-05			
RPS6	5.426835	6.26E-05			
PSMC6	5.1945	0.000172			
ATP2A2	5.166925	0.000172			
HNRNPU	5.010805	0.000266			
